# Predicting Design Solutions with Scenarios Considering the Quality of Materials and Products Based on a Life Cycle Assessment (LCA)

**DOI:** 10.3390/ma17040951

**Published:** 2024-02-19

**Authors:** Dominika Siwiec, Andrzej Pacana

**Affiliations:** Faculty of Mechanical Engineering and Aeronautics, Rzeszow University of Technology, 35-959 Rzeszow, Poland; app@prz.edu.pl

**Keywords:** LCA, quality, vehicle, BEV, ICEV, GRA, mechanical engineering, production engineering

## Abstract

The advancement of quality and environmentally sustainable materials and products made from them has improved significantly over the last few years. However, a research gap is the lack of a developed model that allows for the simultaneous analysis of quality and environmental criteria in the life-cycle assessment (LCA) for the selection of materials in newly designed products. Therefore, the objective of the research was to develop a model that supports the prediction of the environmental impact and expected quality of materials and products made from them according to the design solution scenarios considering their LCA. The model implements the GRA method and environmental impact analysis according to the LCA based on ISO 14040. The model test was carried out for light passenger vehicles of BEV with a lithium-ion battery (LiFePO_4_) and for ICEV. The results indicated a relatively comparable level of quality, but in the case of the environmental impact throughout the life-cycle, the predominant amount of CO_2_ emissions in the use phase for combustion vehicles. The originality of the developed model to create scenarios of design solutions is created according to which the optimal direction of their development in terms of quality and environment throughout LCA can be predicted.

## 1. Introduction

The sustainable improvement of materials and products made from them remains a challenge [1,2]. This is due to the need to meet their expected quality, but also to the needs of environmental protection, such as mitigating climate change, including the need to achieve an economy free of fossil fuels or the need to reduce greenhouse gas (GHG) emissions [3]. New trends force an increase in the quality of materials and products made from them. As part of sustainable processing and production, it is necessary to look for innovative methods to measure their quality and impact on the natural environment [4,5]. This refers to the Sustainable Product Development (SPD) process, where approximately 80% of the total environmental impact is identified [6]. However, this is a difficult and important task in the analyzed research area. This is due to the fact that technology has an impact on the cycle length. Typically, more innovative technologies have a short economic shelf-life [7]. At the early stage of technology development, product innovation activities focus mainly on product quality [8]. Subsequently, for the dominant prototype, market acceptance is predicted and coherence occurs between efforts for product and process innovation [9]. Designing products to be available at scale is key, and this includes technology development. However, such behavior is often accompanied by process rigidity, which limits the scope of further product innovations. In this case, any increase in performance levels results in increased costs, including difficulties in product redesign [10]. Therefore, innovation or technological development is limited [11]. The literature review shows that a life-cycle assessment (LCA) is widely used to analyze various types of materials and products when determining the environmental impact [12].

For example, in the articles [8,13], a biologically based binder was analyzed, which consisted mainly of polylactic acid. The Ti6Al4V raw material suitable for use in metal additive manufacturing was produced. These were comparative analyses according to processes that are applicable to aviation, where the analyses were based on a life-cycle assessment (LCA). However, one study [14] analyzed the production of geopolymer materials in terms of their life-cycle. The results showed that the geopolymer material has a relatively positive impact on the environment, since it produces less carbon dioxide during production compared to traditional concrete. In turn, the study [15] used the life-cycle assessment to estimate the carbon footprint (CF) of hemp-based construction materials. As a result, hemp-based materials have lower greenhouse gas emissions than traditional materials, such as plasterboard. Another example is the elaboration in [16], in which the life-cycle of an industrial light-emitting diode (LED) fixture was assessed, where the analysis included material extraction (raw materials), assembly, distribution, use, and end of life (EoL), including all other emissions. Energy consumption during use has the greatest negative environmental impact. Another example is study [17], which conducted a life-cycle analysis of a polylactic acid (PLA), comparing it with the paper packaging used for the same purpose. PLA packaging has been shown to cause more environmental damage compared to paper packaging. The next example is the article [18], which compares insulating materials made of biological materials. Life-cycle analyses were performed for four biological insulating materials (wood fiber, hemp, linen, and miscanthus) and two nonrenewable insulating materials (Styrofoam and stone wool). Analyses confirmed that biological materials are more environmentally friendly. A similar application of a life-cycle assessment for materials and products made from them is also presented in other works, e.g., [19,20,21,22,23,24]. Based on the literature review, it was concluded that

- A life-cycle assessment (LCA) is used to evaluate various materials and products;
- Various scenarios of production solutions were also analyzed;
- Analyses focused mainly on the environmental performance of materials and products at different phases of their life-cycle;
- There have been no studies in which, for various design scenarios, the quality of materials and products made from them would be predicted, while taking into account their environmental impact throughout the entire life-cycle (LCA).

This was considered a research gap that was intended to be filled by developing a model that supports this process.

The purpose of the research was to develop a model that supports the prediction of the environmental impact and expected quality of materials and products made from them according to the design solution scenarios, taking into account their entire life-cycle (LCA). As part of the investigation, the following hypothesis was adopted:

**Hypothesis** **1.** *A combined assessment of quality and environmental impact throughout the life-cycle for various design solution scenarios will allow for a prediction of the direction of development of materials and products made from them.*

LCA studies, in their inception, are meant to predict environmental impacts. The same applies to environmental impact assessments. Thus, the predictive purpose is clear, but LCA methods are full of failures. Therefore, “predicting” should be understood as the possibility of making some intention to approximate the results within their modeling with them being adopted on the scope and/or scale, which generates different results. In the proposed model, predicting included not only LCA, but also the quality product (aggregate product quality), which refers to different quality criteria and their modification. Therefore, the originality of the research carried out is the development of a model that can be adapted to any materials and products made from them. The universality of the model includes proposing methodology for assessing the quality of materials and products, which is applicable to the analysis of factors in a micro- and multi-criteria approach. At the same time, the model procedure supports carrying out a life-cycle assessment for any selected materials and products. 

The novelty of the developed model concerns the possibility of analyzing variants of the application of different material solutions and products made from them. Based on results from the model, there are scenarios of design solutions. According to which the more possible the most favorable direction of development of products, in terms of simultaneous quality and the environment throughout their entire life-cycle (LCA), can be predicted.

One limitation is access to reliable and complete data on the life-cycle of materials. Additionally, it may be difficult to select a representative and competent group of customers who will be able to precisely comment on the products and the materials used in them. The model supports the methodological and practical integration of LCA with the quality development processes of materials and products. Furthermore, the results obtained can be interpreted as quantitative and qualitative terms, which further increases the effectiveness of the qualitative and environmental assessment evaluation of materials and products.

## 2. Model to Predict Solution Scenarios of the Quality of Materials and Products Based on a Life-Cycle Assessment (LCA)

**Figure 1:**
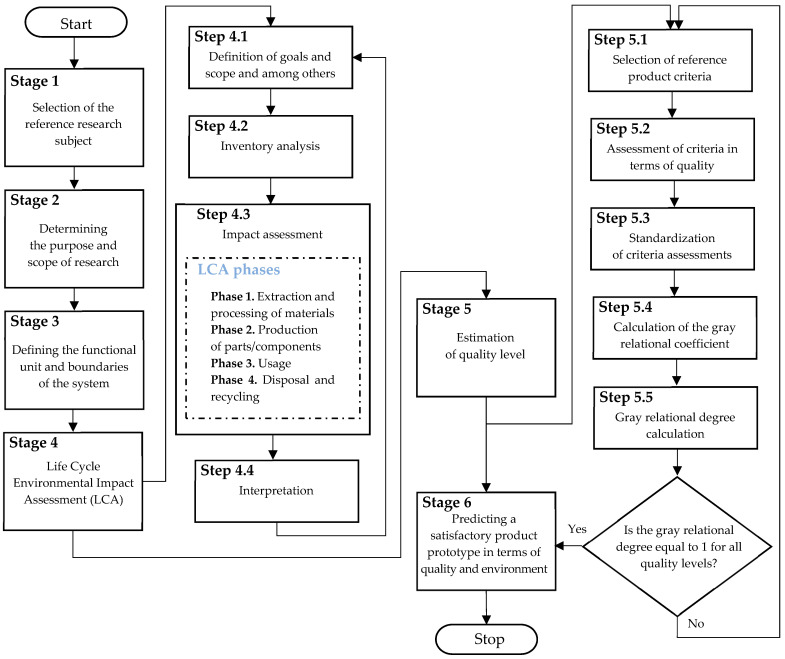
Model supporting the pro-quality improving of materials and products based on a life-cycle assessment (LCA).

The idea of the research is to develop a model for creating design solutions, taking into account the environmental impact throughout the life-cycle and the level of the quality of materials and products made from them [25,26]. The algorithm is shown in Figure 1. 

### 2.1. Stage 1: Selection of the Reference Research Subject

The subject (expert) selects the subject of the research (product). The subject of the research will be analyzed throughout its entire life-cycle, that is, from the mining and extraction of materials to recycling. Additionally, analyses will be carried out on prototypes of the research subject. The mentioned prototypes are alternatives to an existing research subject. These prototypes are also analyzed in terms of the materials they contain throughout their life-cycle. It was assumed that the subject of research is the product of the so-called reference, that is, a generalization of a product of a given type (e.g., for the same purpose). It is possible to select any research subject, e.g., a product in the maturity or decline phase. Based on the analysis of materials and products (including prototypes) created from them, the improvement direction of the existing product will be planned. This prototype will be recommended for production in the final stage of the model.

### 2.2. Stage 2: Determining the Purpose and Scope of Research

The entity (expert) defines the purpose of the research. It is assumed that the aim is to predict the direction of improvement of materials and products made from them according to product prototypes analyzed on the basis of the level of quality and environmental impact throughout the life-cycle (LCA). To precisely define the research goal, the SMARTER method (Specific, Measurable, Achievable, Relevant, based on Timeline) is recommended, which is a method that supports determining the purpose of research, as shown in study [27].

The scope of the investigation includes an analysis of product materials and their prototypes, where the analysis is related to their quality. Qualitative analysis occurs, among others, according to the main materials of the products and then according to the criteria that significantly influence customer satisfaction with usability. Prototypes are created based on modifications to current (existing) product materials and product criteria states. The scope of research also includes an analysis of the product life cycle in order to analyze its environmental impact (e.g., consumption of energy and materials, consumption of carbon dioxide (CO_2_), and emissions of air pollutants and waste).

### 2.3. Stage 3: Defining the Functional Unit and Boundaries of the System

The functional unit and the boundaries of the system are determined by the entity (expert). The functional unit is intended to ensure standardization of the database and a comparison of the materials and products, including the prototypes planned for them. It is a unit of measurement that quantifies the functions of a system. It should be defined in international units of measurement and be adapted to the subject of research. In turn, the model system boundary determines which elements should be included in the investigation. It refers to a temporal and spatial boundary and often depends on the access to data. The system boundary allows you to determine the area of analysis, e.g., referring to inputs and outputs in the process of production and processing, transport, production, and the use of energy, fuels, and heat, or use and recycling, or recovery. The boundaries of the system are set by the expert depending on the needs and available data.

### 2.4. Stage 4: Life-Cycle Environmental Impact Assessment (LCA)

**Figure 2:**
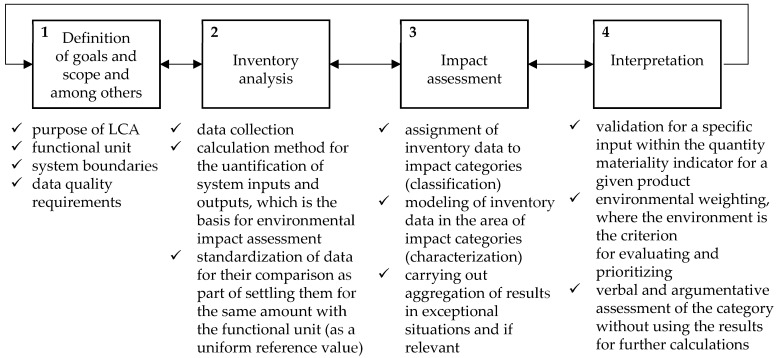
LCA according to ISO 14040. Own study based on [29,30].

The entity (expert) evaluates the environmental impact of the selected reference product throughout its entire life-cycle (LCA), i.e., the existing (current) product. For this purpose, it uses the life-cycle assessment methodology, which is an environmental management method. The assessment covers the environmental hazards of a product or process throughout its entire period of use, i.e., “from cradle to grave”. Therefore, the environmental impact should be estimated throughout the entire life-cycle, that is, taking into account the following phases: extraction and processing of materials, production of parts/components, product use, and recycling. LCA is a systematic method for quantitatively identifying and determining potential environmental loads, where, according to ISO 14040 [28], it is a set of procedures and input data that can be compared with materials and energy along with the environmental impact. The method of performing LCA according to ISO 14040 is shown in Figure 2.

Depending on the needs, the entity (expert) may be supported by programs dedicated to a life-cycle assessment, e.g., SimaPro [31], Gabi [32], OpenLCA [33], or GREET model [34]. A properly conducted life-cycle analysis allows you to make the right decision about the environmental impact, including the selection of a product with the lowest negative environmental impact. 

### 2.5. Stage 5: Estimation of Quality Level

The quality level of a product refers to the satisfaction of the customer with its use. It refers to the phase in the LCA. According to the model concept, the quality level is estimated for prototypes of an already existing product. The goal is to predict the direction of product development to meet customer expectations. Therefore, the criteria for the research subject (reference product) are initially determined [35].

These should be the main criteria (having a significant impact on customer satisfaction with the use of the product). The criteria are selected by the entity (expert) according to the catalogue of the current product (specification) and after preliminary consultation with the client. The number of criteria analyzed should be in the range of 7 ± 2, because, as shown in the literature on the subject, for example [36] this number allows for an effective comparison and analysis of the criteria against each other. All criteria should be characterized according to the assumed parameters for individual product prototypes. This is performed by the entity (expert) taking into account the design assumptions of these prototypes. As a result, a table will be created, including the i-th criteria and their j-th alternatives to design solutions (according to the planned prototypes of the reference product).

This table is used to obtain customer expectations (determine customer satisfaction with the proposed design solutions). For this reason, the matrix is completed by clients who evaluate the criteria and their design alternatives. Grades are awarded on a seven-point grading scale, where 1 is the criterion that is not very satisfactory and 7 is the criterion that is fully satisfactory. Additionally, customers rate the importance of the criteria on the same rating scale, where 1 is the criterion that is not essential, and 7 is the criterion that is definitely the most important. The number of customers from whom expectations are obtained can be estimated according to the method to calculate the research sample, as presented in the literature on the subject [37]. If the number of customers from whom expectations were obtained is greater than 100, then the arithmetic mean is calculated from all scores awarded; otherwise it is the median of the scores [38]. On their basis, the quality level of product prototypes is calculated.

The quality level is estimated according to the GRA (Grey Relational Analysis) method [39]. The choice of this method resulted from its use for analyses in a fuzzy decision-making environment, which corresponds to the model concept in which prototypes of design solutions are analyzed. Furthermore, the GRA method is used to analyze a small number of criteria (even 4 data or more) [40], which is effective due to assumptions regarding the number of criteria to be analyzed (7 ± 2). This improves the efficiency of the developed model and eliminates limitations in the analysis of the criteria for the reference product. 

Therefore, based on the ratings awarded, they are normalized so that the rating values range from 0 to 1. In the GRA method, there may be favorable criteria (i.e., the higher the parameter value, the better) and unfavorable criteria (the higher the parameter value, the worse). Then, Formula (1) is used [40,41]:

(1) $$\{\begin{array}{ccc} x_{i}^{*}\left(k\right)=\frac{x_{i}^{0}\left(k\right)-min\;x_{i}^{0}\left(k\right)}{max\;x_{i}^{0}\left(k\right)-min\;x_{i}^{0}\left(k\right)} & - & beneficial\\ x_{i}^{*}\left(k\right)=\frac{max\;x_{i}^{0}\left(k\right)-x_{i}^{0}\left(k\right)}{max\;x_{i}^{0}\left(k\right)-min\;x_{i}^{0}\left(k\right)} & - & non\text{-}beneficial \end{array}$$

where x and $x_{i}^{0}$ is the original and comparable sequence, *i* is the alternative design solutions, and *k* is the prototype criterion.

Then, the grey relational coefficient is calculated as shown in the Formula (2) [42,43]:

(2) $$\xi_{i}\left(k\right)=\frac{\Delta_{min}+\zeta \Delta_{max}}{\Delta_{0i}\left(k\right)+\zeta \Delta_{max}}$$

where $\Delta_{0i}$ is the deviation sequence, ζ is the distinguished coefficient, which is often assumed as 0.5, and *k* is the criterion.

The mentioned deviation sequence should be calculated according to the relationships given in the Formula (3) [41,43]:

(3) $$\{\begin{array}{c} \Delta_{0i}=\|x_{0}^{*}\left(k\right)-x_{i}^{*}\left(k\right)\|\\ \Delta_{min}=\underset{\forall \;j\in i}{\min}\;\underset{\forall k}{\min}\|x_{0}^{*}\left(k\right)-x_{j}^{*}\left(k\right)\|\\ \Delta_{max}=\underset{\forall \;j\in i}{\max}\;\underset{\forall k\;}{\max}\|x_{0}^{*}\left(k\right)-x_{j}^{*}\left(k\right)\| \end{array}$$

where *k* is the criterion evaluation, and *i*, *j* is 1, 2, …, *n*.

Then, it is possible to calculate the grey relational degree as in Formula (4) [40,42]:

(4) $$\gamma_{i}=\frac{1}{n}\overset{n}{\underset{k=1}{\sum }}\omega_{k}\left(k\right)\;\xi_{i}\left(k\right)=Q_{i}$$

where $\omega_{k}\left(k\right)=1$, $\xi_{i}$ is a grey relational score that shows the level of correlation between the original sequence and a comparable sequence as if they were identical.

It is assumed that the correct result is achieved when the grey relational degree is equal to 1. Otherwise, the procedure for the GRA method should be repeated. In the proposed model, the grey relational degree values (γ) are identified with the quality level of the prototypes of the reference product (*Q*). The higher the *Q* value, the more satisfactory the quality level is for customers. 

### 2.6. Stage 6: Predicting a Satisfactory Product Prototype in Terms of Quality and the Environment

The results of the earlier stages of the model are used to predict a satisfactory product prototype in terms of quality and the environment. This means that at this stage, a prototype (a proposal for improving the existing product) is planned that will meet customer expectations and, at the same time, be environmentally friendly throughout the entire life-cycle. Due to the fact that the quality levels of prototypes are compared with their environmental impact throughout the entire life-cycle, it is necessary to model the change in the LCA value depending on the analyzed product prototype. This is performed according to the Pareto-Lorenz rule, where the change in the environmental impact of the prototype can be modeled based on the environmental impact of the existing product. This change usually occurs by 20% and changes proportionally to the remaining percentage of the total carbon dioxide emissions over the product’s life-cycle. Therefore, to estimate the quality-environmental level of the product and its proposed prototypes, Formula (5) is used:

(5) $$\{\begin{array}{ccc} QLCA=\frac{LCA}{10^{5}}+Q & - & for\;product\\ QLCA_{i}=\left(\%\frac{LCA}{10^{5}}\pm \frac{LCA_{i}}{10^{5}}\right)+Q_{i} & - & for\;prototype \end{array}$$

where *Q* is the quality level, LCA is the environmental impact in LCA, and *i* is 1,2,…, *n*.

A ranking is created based on the estimated QLCA indicators for the product and its prototypes. On its basis, the direction of product improvement is predicted according to product prototypes [44] and analyzed on the basis of the level of quality and environmental impact throughout the life-cycle (LCA). The first position in the ranking is the prototype that most satisfies customers, that is, has the highest possible level of quality and the lowest possible environmental impact throughout its life-cycle. 

## 3. Results

The model test was carried out for light passenger vehicles from a global manufacturer. Light structures are the basic conditions to reduce energy demand and limit the negative impact of vehicles at the stage of their use. Therefore, the production of lightweight materials is a construction method that should be evaluated environmentally as a whole. An effective way to do this is to conduct a life-cycle assessment. The engineering approach to the life-cycle assessment indicates that future changes in these products should also be taken into account in order to predict their environmental impact in advance. This prospective life-cycle assessment allows for the inclusion of future scenarios in the life-cycle assessment. However, this approach is limited. Therefore, a new approach to life-cycle assessment based on solution scenarios, taking into account the quality of materials and products, was tested for commonly used products, such as light passenger vehicles [45]. The test result is presented in six main stages of the model.

### 3.1. Stage 1: Selection of the Reference Research Subject

The analysis was carried out for light passenger vehicles, that is, electric vehicles (BEV, Battery Electric Vehicle) [46] and those with an internal combustion engine (ICEV, Internal Combustion Engine Vehicle) [47]. These vehicles were the subject of research and were considered reference vehicles (a generalization of light passenger vehicles of the mentioned type). Their advantages and disadvantages are presented in the literature on the subject, for example [46,47,48,49,50]. Details of the materials used in this type of vehicles were analyzed at subsequent stages of the model.

### 3.2. Stage 2: Determining the Purpose and Scope of Research

The purpose of the research was to predict the direction of improvement of light passenger vehicles of BEV and ICEV types according to the prototypes proposed for them. the scope of the investigation included the analysis of BEV and ICEV vehicles and the evaluation of their life-cycle (for example, taking into account the consumption of energy and materials, the consumption of carbon dioxide (CO_2_), and the air pollution and waste).

### 3.3. Stage 3: Defining the Functional Unit and Boundaries of the System

The functional unit was 150,000 km travelled by the reference vehicle [50,51,52]. A specific functional unit reduces the analysis of BEV and ICEV vehicles to normalized data, which, according to the overall efficiency of this type of vehicle, is maintained on average at the level of 150,000 km throughout their life-cycle [34,53]. Durability between ICEV and BEV is not equal. Therefore, it is important to remember that the conventional range of 150,000 km traveled by reference vehicles may have different effects on their durability. Also, the accepted value of the distance traveled is not uniformly recognized as 150,000 km. Other authors [54] proposed 200,000 km. Hence, with different model assumptions, different results may be obtained, which will be verified as part of a sensitivity analysis in future studies. A time limit has been established as the system boundary, which concerns the availability of data. The analysis covers data from 2021 to 2023, including data from the GREET v1.3.0.13991 model and data from the customer (customer expectations towards the analyzed reference vehicles). 

### 3.4. Stage 4: Life-Cycle Environmental Impact Assessment (LCA)

**Figure 3:**
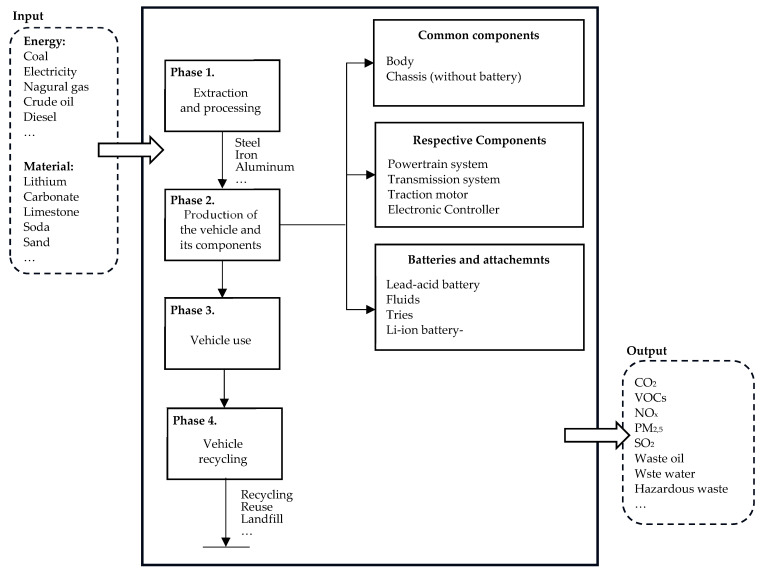
Scheme of vehicle life-cycle analysis. Own study based on [61,62].

The estimation of the environmental impact of vehicles throughout their life-cycle (LCA) was based on data from the GREET v1.3.0.13991 model [34]. The vehicle data modeling method was carried out according to a dedicated method, as presented in the literature on the subject, e.g., [52,55,56,57,58,59,60]. The generalized vehicle LCA analysis scheme is presented in Figure 3.

The entity (expert) assessed the environmental impact of the reference vehicles throughout their life-cycle (LCA). 

#### 3.4.1. Phase 1: Extraction and Processing

This concerns the acquisition and processing of raw materials that are used to build vehicle components. This phase includes, for example, mining, enrichment, smelting, refining, etc., [61]. Carbon emissions from this process were estimated according to Formula (6) [55]:

(6) $$\{\begin{array}{c} C_{M}=\underset{x}{\sum }\left(C_{x,f}+C_{x,e}\right)\\ C_{x,f}=m_{x}\underset{n}{\sum }\left[E_{x,n}\underset{k}{\sum }\omega_{x,n,k}\alpha_{k}\right]\\ C_{x,e,}=m_{x}\underset{n}{\sum }\left(\frac{E_{x,n}\omega_{x,n,e}}{3600}\right) \end{array}$$

where $C_{x,f}$ is the carbon dioxide emissions from fuel consumption during material production, $C_{x,e}$ is the carbon dioxide emissions from electricity consumption during material production, *x* is the material, *m* is the mass (kg), *n* is the production process, $E_{x,n}$ is the energy consumption per unit of material in its production process (kJ/kg), *k* is fuel, $\omega_{x,n,k}$ is the share of fuel consumption in $E_{x,n}$, $\omega_{x,n,e}$ is the share of electricity consumption in $E_{x,n}$, and $\alpha_{k}$ is the fuel carbon emission factor $\left({\mathrm{CO}}_{2}\;\mathrm{kg}/\mathrm{kJ}\right)$.

Therefore, as part of the estimation of carbon emissions from the extraction and processing of materials, a basic list of materials used in the vehicles analyzed was prepared. It was necessary to determine the emission factor during the extraction and processing of these materials. Data acquisition was based on the GREET model and a review of the literature on the subject, for example [56,63,64]. This is presented in Table 1. 

Subsequently, based on data from the GREET model and data from the literature review [55,56], the energy consumption coefficient for the production of materials was determined, and the carbon dioxide emission index was determined for individual types of energy. Coefficients with negligible values were not taken into account (Table 2).

It is necessary to remember that different values of data are given in different sources, as in [54]. This shows that values are subject to variability, among other things, because minerals are produced using different processes based on different ore geologies and ore grades. Then, it was possible to estimate CO_2_ emissions from the extraction and processing of materials for reference vehicles, i.e., electric vehicles (BEV) and combustion engines. Formula (5) was used for this purpose. The results are presented in Table 3. 

The total carbon dioxide emission in the material extraction and processing phase for BEV was $C_{M}$ = 0.29 kWh, while for ICEV, it was $C_{M}$ = 0.27 kWh. Fuel emissions during material extraction and processing were found to contribute more than energy emissions for both BEV and ICEV reference vehicles. This analysis ignores the extraction and processing of materials, including the battery. Then, it was shown that carbon dioxide emissions in this process are slightly higher for reference BEV vehicles.

#### 3.4.2. Phase 2: Production of Vehicle Components

This phase includes the production of vehicle components, where the analysis mainly takes into account the components. Emissions arising from the processing of these components and their installation in the vehicle, e.g., turning, welding, and painting, are taken into account. Distribution/transport can be included in this phase [61]. CO_2_ emissions during the vehicle and its components are calculated according to Formula (7) [55]:

(7) $$\{\begin{array}{c} C_{VA}=\underset{x}{\sum }\left(C_{y,f}+C_{y,e}\right)+\frac{E_{VA}}{3600}\\ C_{y,f}=\underset{q}{\sum }\left[E_{y,q}\underset{k}{\sum }\omega_{y,q,k}\alpha_{k}\right]\\ C_{y,e,}=\underset{q}{\sum }\left(\frac{E_{y,q}\omega_{y,q,e}}{3600}\right) \end{array}$$

where $C_{y,f}$ is the carbon dioxide emissions from fuel consumption based on the production of the component, $C_{y,e}$ is the carbon dioxide emissions from the consumption of electricity based on the production of the component, y is the component (part) of the vehicle, $E_{VA}$ is the electricity consumption during vehicle assembly, *q* is the production process, $E_{y,q}$ is the energy consumption of a component in the production process (kJ), $\omega_{y,q,k}$ is the share of fuel consumption in $E_{y,q}$, $\omega_{y,q,e}$ is the share of electricity in $E_{y,q}$, and $\alpha_{k}$ is the fuel carbon emission factor $\left({\mathrm{CO}}_{2}\mathrm{kg}/\mathrm{kJ}\right)$.

According to data from the GREET model and based on a review of the literature on the subject [55,65,66,67,68] electricity consumption and carbon dioxide emissions were determined for reference vehicles (with an average weight of 1532 kg). The results are shown in Table 4.

In the case of electric vehicles (BEVs), the essential element is the battery. It contains a significant number of rare-earth elements. These elements have high economic value and high rare earth value. The LFP battery (lithium ion, lithium ion) consists of the following:

- Cathode—the main material is LiFePO_4_, the production of one gram of LiFePO_4_ requires 0.23 g of LiCO_3_ and the consumption of 3 kJ of electricity;
- Anodes—their production requires graphite coated with copper foil and a binder;
- Separator—polypropylene and polyethylene;
- Electrolyte—mainly consisting of lithium hexafluorophosphate and dimethyl carbonate;
- Packaging—made of polypropylene and aluminum foil;
- Battery management system—wire, printed circuit board, and sensor;
- According to the GREET model and on the basis of the literature on the subject [48,59,69], a list of materials for a lithium ion battery (used in a reference electric vehicle) was developed. This is presented in Table 5.

According to data from the GREET model and according to the review of the literature on the subject [46,55,70,71,72], a list of energy consumption of lithium-ion batteries was developed, as shown in Table 6.

According to the study [55], the energy consumption when installing this battery in a light passenger vehicle is 2.67 MJ/kg, i.e., in the case of the weight of the analyzed battery, it is approximately 1002 MJ. However, the electricity consumption during the assembly of the remaining vehicle components (that is, without batteries) is 862 MJ. Based on Formula (7), the total emissions during the production of reference vehicles were calculated. Then, for BEVs (with a battery), the energy consumption is $C_{VA}\;$= 9985.34 kWh. For ICEV, the energy consumption is $C_{VA}\;$= 10,075.97 kWh. It has been observed that in this phase, energy consumption is higher in the case of BEVs because of the battery present in them.

#### 3.4.3. Phase 3: Usage

This refers to energy consumption and carbon emissions during vehicle use. In this phase, fuel consumption and vehicle maintenance are also taken into account [61]. Formula (8) is used to calculate these emissions [55]:

(8) $$\{\begin{array}{ccc} C_{VU,EV}=\frac{dP_{E}}{100C_{E}} & - & for\;electric\;vehicles\\ C_{VU,ICEV}=\frac{dF_{k}}{100}\left(\rho_{k}\alpha_{k}LHV_{k}+C_{k}\right) & - & for\;vehicles\;with\;combustion\;engine \end{array}$$

where *P_E_* is the electricity consumption per 100 km by the electric vehicle (kWh/km), *C_E_* is the charging efficiency, *d* is the total driving distance of the vehicle (km), *F_k_* is the fuel consumption per 100 km for a vehicle with an internal combustion engine (l), $\rho_{k}$ is the fuel density, *k* is the fuel, $LHV_{k}$ is the lower thermal value of the fuel (kJ/kg), $\alpha_{k}$ is the fuel carbon emission factor $\left({\mathrm{CO}}_{2}\;\mathrm{kg}/\mathrm{kJ}\right)$, and *C_k_* is the carbon mission per unit *k* in fuel production.

Based on data characterizing the reference vehicles, it was possible to determine emissions during their use. The selected data are presented in Table A1.

According to the adopted data, the energy consumption and CO_2_ emissions were estimated throughout the trip of the reference vehicles. It was assumed that the mileage of the vehicle is 150,000 km during its life-cycle. Therefore, electric vehicles (BEV) under the given assumptions emit $C_{VU}$ = 27,574 kWh during their use. In turn, internal combustion vehicles (ICEV) emit $C_{VU}$ = 506,632 kWh under the given assumptions. In the case analyzed, much higher CO_2_ emissions are generated when using combustion vehicles compared to electric vehicles.

#### 3.4.4. Phase 4: Recycling

In this phase, the emissions generated during the recycling of selected elements are analyzed, including the dismantling of vehicle components. Metals and other nonmetallic materials are separated and purified. It is possible to divide this phase into metal recycling without batteries and metal and battery recycling. Other materials, such as glass and plastic, are stored or burnt. Therefore, the recycling phase also includes disposal and reuse [61]. To estimate carbon dioxide emissions in vehicle recycling, we use Formula (9) [55]:

(9) $$\{\begin{array}{c} C_{RE}=C_{re,f}+C_{re,e}\\ C_{re,f}=\underset{x}{\sum }\left[m_{x}E_{re,x}\underset{k}{\sum }\left(\omega_{re,x,k}\alpha_{k}\right)\right]\\ C_{re,e}=\left[\frac{E_{vd}}{3600}+\underset{x}{\sum }\left(m_{x}\frac{E_{re,x}\omega_{re,x,e}}{3600}\right)\right] \end{array}$$

where $C_{re,f}$ is the carbon dioxide emissions from fuel consumption in vehicle recycling, $C_{re,e}$ is the carbon dioxide emissions from electricity consumption in vehicle recycling, $E_{re,x}$ is the energy consumption per unit of material *x* in the recycling phase (kJ/kg), *x* is the recycled material, $\omega_{re,x,k}$ is the share of fuel consumption in $E_{re,x}$, $\omega_{re,x,k}$ is the share of electricity consumption in $E_{re,x}$, m is the mass (kg), and $E_{vd}$ is the energy consumption when dismantling the vehicle.

According to the given formula, emissions were calculated during the recycling of selected components of reference vehicles of the BEV type (taking into account battery recycling using hydrometallurgical technology) and the ICEV type. It was based on data from the GREET model and a literature review and reports [73,74,75,76], as show in Table 7.

Carbon dioxide emissions in the recycling phase of a BEV vehicle (taking battery recycling into account battery recycling) amounted to $C_{RE}$ = 1898.04 kWh, where excluding battery recycling it was $C_{RE}$ = 1760.22 kWh. However, during the recycling of ICEVs, emissions were estimated at the level of $C_{RE}$ = 1820.71 kWh This is a smaller amount of emissions compared to recycling an electric vehicle with a battery. Battery recycling has been shown to emit a significant amount of CO_2_ in the last phase of LCA, as also confirmed by the authors of the studies [73,76,77].

Based on the calculated carbon dioxide emission rates in individual phases of the vehicle life-cycle, it is possible to calculate their total emissions. For this purpose, Formula (10) is used [55]:

(10) $$LCA=C_{M}+C_{VA}+C_{VU}+C_{RE}$$

where LCA is the total carbon dioxide emissions over the vehicle’s life-cycle, $C_{M}$ is the carbon emissions from the extraction and processing of the material, *C_VA_* is the carbon dioxide emissions from the production of the vehicle and components, *C_VU_* is the carbon dioxide emissions from vehicle use, *C_RE_* is the carbon dioxide emissions in vehicle recycling.

The total environmental impact of the BEV reference vehicle was estimated to be LCA = 39,457.66 kWh, while for the ICEV reference vehicle it was an ICEV with LCA = 518,528.95 kWh. The negative environmental impact over the entire life cycle of ICEV vehicles has been shown to be higher by 479,071.29 kWh compared to the impact of BEV vehicles. The biggest differences occur in the vehicle phase, where combustion vehicles emit much larger amounts of carbon dioxide compared to electric vehicles.

### 3.5. Stage 5: Estimation of Quality Level

As part of the evaluation of the quality level of reference vehicles, the criteria (attributes) of these vehicles that have a significant impact on customer satisfaction with the vehicle use of vehicles. The criteria were selected by the entity (expert) based on the catalogue of reference vehicles and were as follows: dimensions, kerb weight, maximum engine power, maximum speed, and color of the car. According to the assumptions adopted, the number of criteria was in the range of 7 ± 2 [36]. Based on current vehicle criteria, their possible modifications (so-called vehicle prototypes) were developed. The prototypes were planned according to the Pareto principle (20/80) [78], where the reference vehicle criteria were modified by 20% relative to the current criterion parameter, as shown in Table A2. According to the general description of the model (stage 5, Section 2), the quality level of the vehicles was estimated according to the GRA method. The results are presented in Table A3, Table A4, and Table 8.

In the proposed model, the values of the grey relational degree (γ) are identified with the quality level of the prototypes of the reference product (Q). In the example analyzed, the most advantageous were the second and third prototypes of the BEV vehicle, as well as the third prototype of the ICEV vehicle. If the decision on the direction of improvement was made only with quality in mind, then based on these results, improvement actions would have to be taken to achieve the quality level of the first-ranked prototypes.

### 3.6. Stage 6: Predicting a Satisfactory Product Prototype in Terms of Quality and the Environment

The results of the earlier stages of the model were used to predict customer satisfaction with the current BEV and ICEV reference vehicles and their proposed prototypes in terms of quality and the environment. For this purpose, Formula (5) from the model was used, where it was assumed that the change in environmental impact throughout the life-cycle of the prototypes occurs by 20% in proportion to the estimated environmental impact for the reference vehicle. The result is shown in Table 9.

**Figure 4:**
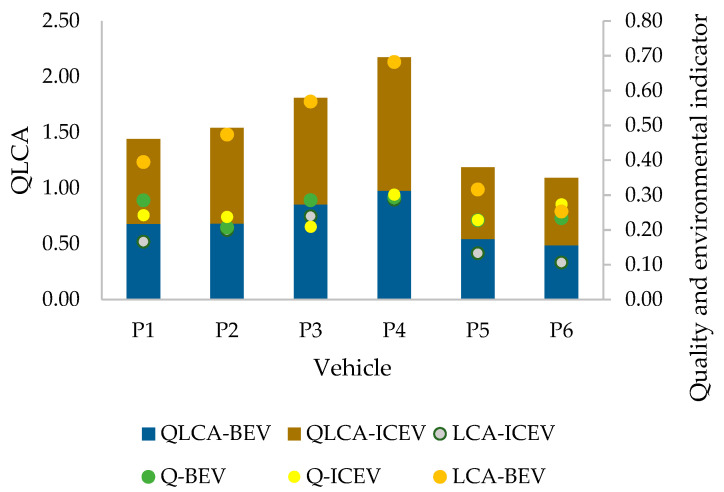
Comparison results of scenarios obtained using the developed model.

Based on the analysis of the model results, it was concluded that in this case, the quality levels of BEVs and ICEVs were relatively similar. This resulted from the individual preferences of the customer who participated in the study. However, the environmental impact of vehicles throughout their life-cycle varied significantly and contributed significantly to the final QLCA, as Figure 4 shows.

This is a situation where the level of quality and environmental impact are assumed to be equivalent. However, if this importance was different, the model’s results regarding the final QLCA index could change significantly. Hence, the model is effective in analyzing the quality level of materials and products made from them and supports their assessment throughout their life-cycle (LCA). Based on the results obtained, it is possible to predict the direction of improvement of materials and products according to their proposed prototypes.

## 4. Discussion

The sustainable improvement of materials and products made from them concerns their quality and environmental impact. Competent responses to climate change mean that this process often covers the entire life cycle (LCA) [80]. However, it is difficult to dynamically respond to customer needs, including environmental changes. Therefore, various methods and tools supporting the sustainable design of materials, products, and processes are being sought [81,82].

Therefore, the objective of the research was to develop a model that supports the prediction of the environmental impact and expected quality of materials and products made from them according to design solution scenarios, taking into account their entire life-cycle (LCA).

The main benefits of the proposed model include the following:

- Determining the current and expected level of quality of materials and products made from them, including their prototypes according to alternative criteria and their modifications;
- Estimation of the environmental impact throughout the life-cycle of any reference products;
- Taking into account customer expectations in the product-improvement process;
- Predicting the quality of product prototypes and the environmental impact throughout the life-cycle (LCA) of materials and products made from them, including prototypes modeled according to design solution scenarios.

However, the limitations of the model are the need for knowledge and the ability to assess the product life-cycle by the entity (expert) using the model. Depending on the needs of the entity, this assessment is for the selected research subject. Another limitation of the model is the need to adjust the research sample to precisely predict actions to improve a given type of product.

Results of the model confirmed the assumed hypothesis that a combined assessment of quality and environmental impact throughout the life-cycle for various design solution scenarios will allow for a prediction of the direction of development of materials and products made from them.

In future research, it is planned to analyze other types of materials and products to assess their environmental impact throughout their life-cycle and expand the proposed model with additional modules. Future research will aim to expand the model to include cost aspects so that improvement decisions are based simultaneously on the three pillars of sustainable development. This resulted from the fact that maintenance costs may be different from traditional engines with respect to electric ones. Therefore, it is an important criterion to have a coherent and complex analysis.

## 5. Conclusions

The research included a prospective approach to a life-cycle assessment (LCA) and an assessment of the quality level of materials and products made from them. The aim of the research was to develop a model that supports the prediction of the environmental impact and expected quality of materials and products made from them according to the design solution scenarios, taking into account their entire life-cycle.

The model was created in five main stages, and its test was carried out for light passenger vehicles of the BEV type with a lithium-ion battery (LFP, LiFePO_4_) and for the ICEV (Internal Combustion Engine Vehicle). The quality level of these vehicles and their prototypes was estimated according to Grey Relational Analysis (GRA), where the analyzed criteria were the dimensions, kerb weight, maximum engine power, maximum speed, and car color. After the quality level, it was shown that in this case, the quality of BEVs and ICEVs was relatively similar. Then, a life-cycle assessment of these vehicles was carried out. The total environmental impact of the BEV-type reference vehicle has been shown to be LCA = 39,457.66 kWh, while for the ICEV-type reference vehicle, it is LCA = 518,528.95 kWh. It was concluded that the greatest differences occur in the vehicle-use phase, where combustion vehicles emit much higher amounts of carbon dioxide compared to electric vehicles. Based on the overall analysis (taking into account the quality level and environmental impact) and according to the design solution scenarios, the most advantageous vehicle prototype was identified.

Some limitation is access to reliable and complete data on the life-cycle of materials. Additionally, it may be difficult to select a representative and competent group of customers who will be able to precisely comment on the products and the materials used in them. Also, load-bearing or heavy machines and materials have a short life-cycle, which makes it easy for the model to be examined.

The model allows for an effective methodological and practical integration of LCA with the quality development processes of any material and products. For this reason, it can be used to analyze various design solutions of materials and products to predict the optimal direction of their development.

## Figures and Tables

**Table 1:** Main materials for light passenger vehicles.

Material	Mass of Main Materials (kg)		Emission Factor of Material Production (kg/kg)
	BEV	ICEV	
Steel	831	793	2.00
Iron	31	139	0.55
Cast aluminum	12	28	2.62
Wrought aluminum	78	59	5.92
Copper	38	24	2.35
Glass	46	37	1.62
Plastic	155	141	3.05
Rubber	21	29	3.62

**Table 2:** Energy consumption factor for material production and CO_2_ emission factor (excluding battery).

Fuel	Energy Consumption for the Production of Materials (MJ/kg)						
	Coal	Natural Gas	Crude Oil	Coke	Gasoline	Electricity	Diesel
Indicator emissions CO_2_	0.10	0.06	0.11	0.08	0.09	0.19	0.09
Steel	2.13	0.83	1.21	0.03	0.00	0.20	0.00
Iron	0.00	0.57	0.26	0.02	0.00	0.07	0.00
Aluminum	5.04	0.80	0.00	0.00	0.00	1.54	0.00
Copper	4.78	0.57	0.00	0.00	0.00	1.47	0.00
Glass	0.44	0.00	0.00	0.68	0.00	0.55	0.96
Plastic	0.32	1.61	0.00	0.03	0.00	0.10	0.00
Rubber	0.04	1.98	0.00	0.36	0.01	0.15	0.02

**Table 3:** Carbon dioxide emissions from the extraction and processing of materials for BEV and ICEV reference vehicles.

Material	$C_{x,f}$		$C_{x,e}$		$C_{M}$	
	BEV	ICEV	BEV	ICEV	BEV	ICEV
Steel	654.05	624.31	0.09	0.09	654.15	624.39
Iron	1.16	5.13	0.00	0.00	1.16	5.14
Aluminum	17.85	39.86	0.01	0.03	17.86	39.89
Copper	233.12	175.69	0.19	0.14	233.30	175.83
Glass	9.02	5.76	0.01	0.01	9.03	5.76
Plastic	10.25	8.28	0.00	0.00	10.25	8.28
Rubber	76.69	69.48	0.02	0.02	76.71	69.50

Where: $C_{x,f}$—carbon dioxide emissions from fuel consumption during material production, $C_{x,e}$—carbon dioxide emissions from electricity consumption during material production, $C_{M}$—carbon emissions from extraction and processing of the material.

**Table 4:** Energy consumption and carbon dioxide emissions from the production process of the components of the reference passenger vehicle (excluding the battery).

Process	Energy Consumption [MJ/kg]	CO_2_ Emission [kg/kg]
Stamping	5.1	0.31
Shape casting aluminum	55.3	3.08
Iron	32	1.69
Copper wire production	7.1	0.43
Brass from scrap	7.4	0.42
Secondary lead production	8.5	0.49
Machining	2.015	0.115
Forging	45.1	2.61
Glass pane forming	16	0.93
Welding	920	62
Painting	4167	268
HVAC and lighting	3335	225
Material handling	690	39.5
Heating	3110	195
Compressed air	1380	93
Moldings–Rubber	12.9	0.74
Moldings–Thermosets	4.79	0.27
Injection mold–PP	26.4	1.53
Injection mold–PVC	24.3	1.56
Blow mold HDPE	19.7	1.13
Calendaring PVC Sheet	6.25	0.36
Extrusion HDPE pipe	7.03	0.42

**Table 5:** Bill of materials for lithium-ion battery.

Material	LFP Battery (kg)
Steel	4.08
Aluminum	134.49
Copper	36.68
Plastic	28.53
LiFePO4	89.66
Graphite	40.76
Electrolyte	40.76

**Table 6:** Energy consumption inventory of the lit-on battery (MJ/kWh).

Component	Electricity	Coal	Crude Oil	Natural Gas
Cathode	0.02	0.14	0.02	0.44
Anode	0.00	0.10	0.02	0.54
Separator	0.00	0.02	0.00	0.02
Electrolyte	112.34	0.00	0.00	0.00
Packaging	2.40	23.40	0.56	33.20
BMS	5.74	0.00	0.00	0.00
Battery package	147.00	0.00	0.00	0.00

**Table 7:** Energy consumption in the recycling phase of reference vehicles including the lithium-ion battery.

Process	BEV			ICEV		
	Electricity (kWh)	Natural Gas (kWh)	Coal (kg)	Electricity (kWh)	Natural Gas (kWh)	Coal (kg)
Vehicle assembly	627.3	-	-	618.08	-	-
Non-battery parts	1114	83.36	9.79	1170.8	11.19	20.64
Lit-on accumulator	62.26	1.33	-	-	-	-

**Table 8:** GRA method results determining the quality level of reference vehicles [79].

Criteria	C1	C2	C3	C4	C5	Q	Ranking	Criteria	C1	C2	C3	C4	C5	Q	Ranking
Weights	0.40	0.40	0.60	0.70	0.50			Weights	0.40	0.40	0.60	0.70	0.50		
BEV	0.27	0.40	0.24	0.30	0.50	0.28	2	ICEV	0.22	0.40	0.24	0.23	0.36	0.24	3
P1	0.20	0.20	0.30	0.35	0.19	0.21	4	P1	0.18	0.20	0.24	0.30	0.50	0.24	3
P2	0.13	0.40	0.40	0.42	0.36	0.29	1	P2	0.13	0.13	0.40	0.42	0.17	0.21	5
P3	0.16	0.13	0.60	0.70	0.17	0.29	1	P3	0.18	0.13	0.60	0.70	0.19	0.30	1
P4	0.20	0.20	0.20	0.26	0.50	0.23	3	P4	0.18	0.40	0.20	0.42	0.17	0.23	4
P5	0.40	0.40	0.20	0.23	0.17	0.23	3	P5	0.40	0.20	0.24	0.30	0.50	0.27	2

Where: P1–P5—prototype.

**Table 9:** The expected direction of improvement of reference vehicles in terms of quality and the environment throughout their life-cycle.

Prototypes	Q		LCA		BEV		ICEV	
	BEV	ICEV	BEV	ICEV	QLCA	Ranking	QLCA	Ranking
Current Vehicle	0.28	0.24	0.39	0.52	0.68	3	0.76	3
Prototype 1	0.21	0.24	0.47	0.62	0.68	3	0.86	4
Prototype 2	0.29	0.21	0.57	0.75	0.85	4	0.96	5
Prototype 3	0.29	0.30	0.68	0.90	0.98	5	1.20	6
Prototype 4	0.23	0.23	0.32	0.41	0.54	2	0.64	2
Prototype 5	0.23	0.27	0.25	0.33	0.49	1	0.61	1

## Data Availability

The data presented in this study are available on request from the corresponding author.

## Appendix A

**Table A1:** Consumption data for reference vehicles.

**BEV**	
*d* (km)	531
*P_E_* (kWh/km) per 100 km traveled	17.28
*C_E_* (%)	94
**ICEV**	
*d* (km)	531
*F_k_* (l) per 100 km traveled	4.6
$\rho_{k}$ (kg)	0.75
$\alpha_{k}$ $\left({\mathrm{CO}}_{2}\mathrm{kg}/\mathrm{kJ}\right)$	2.31
${LHV}_{k}$ (kJ/kg)	42
*C_k_* (kg)	0.66

**Table A2:** Characteristics of reference vehicles and proposed prototypes of these vehicles.

**Criterion**	**BEV**	**Prototype 1**	**Prototype 2**	**Prototype 3**	**Prototype 4**	**Prototype 5**
C1	4749 × 2125 × 1622	5699 × 2550 × 1946	6839 × 3060 × 2335	8207 × 3672 × 2802	3799 × 1700 × 1298	3039 × 1360 × 1038
C2	1996	1597	1916	2299	1597	1278
C3	344	413	496	595	275	220
C4	240	288	346	415	192	154
C5	black	white	silver	red	black	blue
**Criterion**	**ICEV**	**Prototype 1**	**Prototype 2**	**Prototype 3**	**Prototype 4**	**Prototype 5**
C1	4595 × 1890 × 1401	5514 × 2268 × 1681	6617 × 2722 × 2017	7940 × 33,266 × 2420	3676 × 1512 × 1121	2941 × 1210 × 897
C2	1429	1715	2058	2470	1976	1581
C3	329	395	474	569	455	364
C4	275	330	396	475	380	304
C5	silver	black	red	white	blue	back

Where: C1—dimensions (length/width/height) (mm), C2—own weight (kg), C3—max. engine power, C4—max. speed (km/h), C5—car color.

**Table A3:** Criterion ratings of reference vehicles assigned by the customer according to the GRA method.

Criteria	C1	C2	C3	C4	C5	Criteria	C1	C2	C3	C4	C5
Kind of criteria	Cost	Cost	Beneficial	Beneficial	Beneficial	Kind of criteria	Cost	Cost	Beneficial	Beneficial	Beneficial
Weights	0.40	0.40	0.60	0.70	0.50	Weights	0.40	0.40	0.60	0.70	0.50
BEV	4	4	4	3	7	ICEV	4	4	4	4	6
Prototype 1	5	5	5	4	3	Prototype 1	5	5	4	5	7
Prototype 2	7	4	6	5	6	Prototype 2	7	6	6	6	2
Prototype 3	6	6	7	7	2	Prototype 3	5	6	7	7	3
Prototype 4	5	5	3	2	7	Prototype 4	5	4	3	6	2
Prototype 5	3	4	3	1	2	Prototype 5	2	5	4	5	7

**Table A4:** Grey relational factor in the GRA method for calculating the quality of reference vehicles.

Criteria	C1	C2	C3	C4	C5	Criteria	C1	C2	C3	C4	C5
Kind of criteria	Cost	Cost	Beneficial	Beneficial	Beneficial	Kind of criteria	Cost	Cost	Beneficial	Beneficial	Beneficial
Weights	4	4	6	7	5	Weights	4	4	6	7	5
BEV	0.67	1.00	0.40	0.43	1.00	ICEV	0.56	1.00	0.40	0.33	0.71
Prototype 1	0.50	0.50	0.50	0.50	0.38	Prototype 1	0.45	0.50	0.40	0.43	1.00
Prototype 2	0.33	1.00	0.67	0.60	0.71	Prototype 2	0.33	0.33	0.67	0.60	0.33
Prototype 3	0.40	0.33	1.00	1.00	0.33	Prototype 3	0.45	0.33	1.00	1.00	0.38
Prototype 4	0.50	0.50	0.33	0.38	1.00	Prototype 4	0.45	1.00	0.33	0.60	0.33
Prototype 5	1.00	1.00	0.33	0.33	0.33	Prototype 5	1.00	0.50	0.40	0.43	1.00
